# Effects of small-sided recreational team handball training on mechanical muscle function, body composition and bone mineralization in untrained young adults—A randomized controlled trial

**DOI:** 10.1371/journal.pone.0241359

**Published:** 2020-11-18

**Authors:** Bjørn Fristrup, Peter Krustrup, Jesper L. Andersen, Therese Hornstrup, Frederik T. Løwenstein, Mikkel A. Larsen, Jørn W. Helge, Susana C. A. Póvoas, Per Aagaard

**Affiliations:** 1 Department of Sports Science and Clinical Biomechanics, SDU Sport and Health Sciences Cluster (SHSC), University of Southern Denmark, Odense, Denmark; 2 Institute of Sports Medicine Copenhagen, Bispebjerg Hospital, University of Copenhagen, Copenhagen, Denmark; 3 Department of Nutrition, Exercise and Sports, University of Copenhagen, Copenhagen, Denmark; 4 Center for Healthy Aging, Department of Biomedical Sciences, University of Copenhagen, Copenhagen, Denmark; 5 Sport and Health Sciences, Faculty of Life and Environmental Sciences, University of Exeter, Exeter, United Kingdom; 6 Shanghai University of Sport (SUS), Shanghai, China; 7 Research Center in Sports Sciences, Health Sciences and Human Development, CIDESD, University Institute of Maia, ISMAI, Maia, Portugal; 8 Department of Sports Science and Clinical Biomechanics, Muscle Physiology and Biomechanics, University of Southern Denmark, Odense, Denmark; The University of British Columbia, CANADA

## Abstract

Prolonged physical inactivity in young adults may lead to deficiencies in musculoskeletal fitness, and thus a need exists to develop physical activity and exercise programmes that are effective of increasing musculoskeletal fitness. The aim of this study, therefore, was to investigate the effects of small-sided team handball training on lower limb muscle strength, postural balance and body composition in young adults. Twenty-six men and twenty-eight women were stratified for peak oxygen uptake (VO_2peak_) and body fat percentage and randomly allocated to either 12 wks of small-sided recreational team handball training (THG: 14 men and 14 women, age 24.1±2.6 yrs (mean±SD), VO_2peak_ 39.8±5.9 ml/kg/min and body fat percentage 32.7±8.7%) or serving as non-exercising controls (CON: 12 men and 14 women, age 24.8±3.1 yrs, VO_2peak_ 39.7±5.0 ml/kg/min, body fat percentage 31.7±9.7%). THG trained on average 1.8 times/week for 12 wks. At 0 and 12 wks, lower limb muscle strength, rate of force development (RFD), vertical jump height and power, postural balance, body composition and muscle biopsies were assessed. No training effects were observed for maximal isokinetic or isometric knee extensor strength, maximal vertical jump height or take-off power, fibre type distribution or capillarization. Late phase (RFD) increased (+7.4%, *p*<0.05) and postural sway excursion length was improved after training (-9%, *p*<0.05) in THG with no difference from CON (*p*>0.05). Further, THG demonstrated a decrease in body fat percentage (-3.7%) accompanied by increases in whole-body fat free mass (FFM) (+2.2%), leg FFM (+2.5%), total bone mineral content (BMC) (+1.1%), leg BMC (+1.2%), total hip bone mineral density (+1.6%) and hip T-score (+50%) which differed from CON (all *p*<0.05). In conclusion, recreational small-sided team handball training appears to effectively improve rapid force capacity, postural balance, lean and fat body mass and bone health in previously untrained young adults. The study was registered at ClinicalTrials.gov (NCT04247724).

**ClinicalTrials.gov ID number:** NCT04247724

## Introduction

Insufficient levels of physical activity is among the leading risk factors for non-communicable diseases such as poor musculoskeletal fitness, metabolic- and cardiovascular diseases [1–3]. Insufficient musculoskeletal fitness has been correlated to low muscle strength, sarcopenia, poor postural balance, osteopenia, and osteoporosis [4–6] and consequently, it becomes of importance to develop exercise programs aiming to increase physical activity levels. It has been stated, that participation in regular physical activity from a young age will increase the chance for continuing physical activity across a lifespan [7] and thereby reduce the risks associated with physical inactivity.

Small-sided recreational football training has proven beneficial for improving cardiovascular, metabolic and musculoskeletal fitness for various population groups [8–10]. Specifically, maximal dynamic [11] and isometric muscle strength [12], vertical jump height [11, 12] and lower limb muscle power [11] as well as postural balance [13, 14] were all enhanced with training. In addition, body fat percentage decreased [15], while fat free mass (FFM) [16–19] and leg FFM [19] increased along with signs of enhanced bone health [11, 18], following small-sided football fitness training. Further increases in muscle fiber cross sectional area (CSA) [14, 17], and reduced percentage of type IIx muscle fibres with a tendency for elevated type IIa [17, 20] have been reported following training. Therefore, it was of interest to investigate if other modified versions of known intermittent sports could elicits same improvements of the musculoskeletal fitness.

It has been suggested that the many intense actions during small-sided recreational football are the main cause for the observed positive adaptations in musculoskeletal function [10, 21–23]. Notably, recreational small-sided team handball match-play has shown similar patterns of intermittent movements and activity changes as reported in small-sided football play [21, 24–26]. Yet, only few studies have investigated the physical adaptations to small-sided team handball training with no investigations into the potential effect of small-sided team handball training on musculoskeletal fitness.

Based on previous study reports on the physiological effects of small-sided intermittent football training, it was therefore our hypothesis that the many intense actions and changes of direction performed during small-sided team handball would lead to improvements in lower limb muscle strength, muscle power, muscle endurance and bone health in untrained adults, compared to non-exercising controls.

The primary outcome variables of the present study were maximal dynamic (isokinetic) and static (isometric) muscle strength and power of the knee extensors. As a secondary outcome, the study also intended to investigate the effects of small-sided team handball on postural balance, body composition and performance measures such as maximal vertical jump height. Lastly, muscle fiber composition was examined as well.

## Material & methods

### Study design

This study was a part of a larger study, investigating the cardiovascular effects of small-sided team handball for untrained men and women. The study took place from October to December 2015. The primary outcome variable of the overall study was VO_2peak_, as described by Hornstrup and colleagues [24, 25]. Sample size estimates were based on the expected standard deviations of changes in the main outcomes of VO_2peak_ and body-composition previously reported following recreational small-sided football training in untrained individuals [18]. An expected completion of 75% of the enrolled participants led to include a minimum 64 study participants. The study was set up as a randomized controlled trial and in this sub study we aimed at investigating the concurrent changes in mechanical muscle function and physical performance elicited by this intervention.

### Study participants

**Fig 1:**
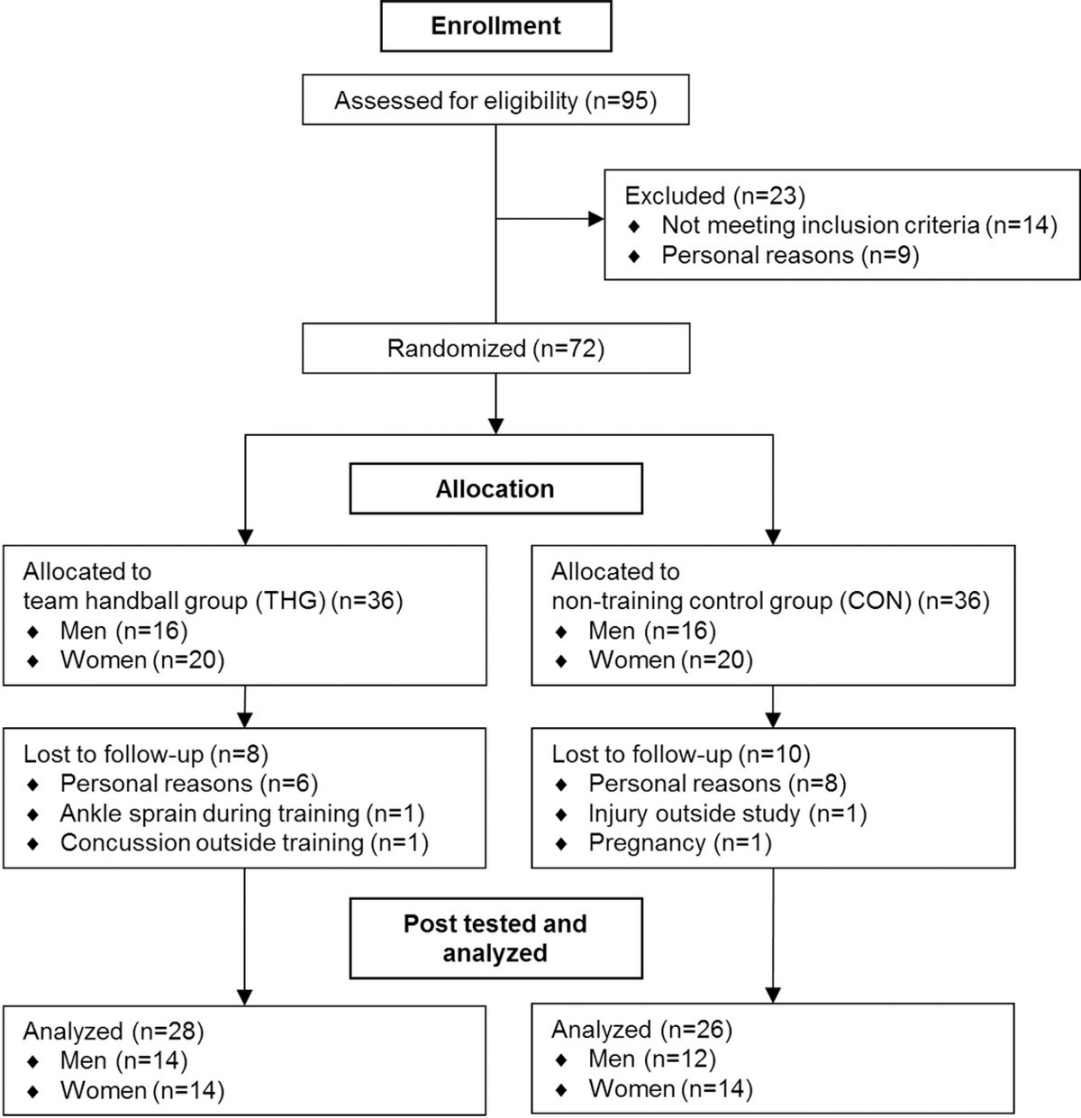
Participant recruitment. Flow diagram depicting the inclusion/exclusion of study participants in the initial phase of enrolment and during the subsequent phases of intervention allocation, post testing, and data analysis.

Ninety-five healthy men and women were recruited trough advertising in local newspapers and on social media. Prior to group allocation, all study participants performed an incremental treadmill test to exhaustion for VO_2peak_ assessment (OxyconPro; Viasys Healthcare, Hoechberg, Germany) along with measurement of body height and body mass (Seca 220, Vokel & Halke, Hamborg, Tyskland) (Table 1). To meet the inclusion criteria, participants were not allowed to have any known family diseases, nor prior participation in regular exercise activity for the past two years (biking for commuting allowed). To avoid inclusion of well-trained or highly trained subjects, study participants were required to have VO_2peak_ values <51 ml/min/kg (males) and <45 ml/min/kg (females). Thirty-two men and forty women met the defined criteria and were included in the study. The study participants were stratified for gender, VO_2peak_ and body fat percentage and randomly (dichotomic ballot drawing) allocated to either a team handball training group (THG) or serving as non-training controls (CON). When two study participants were matched on gender, VO_2peak_ and body fat percentage, the ID number of each study participant, respectively, were put in opaque envelopes and drawn from sealed containers for HTG or CON allocation. After completion of the randomization process, group allocation was revealed for both scientists and subjects. One study participant from THG had to withdraw from the study, due to an ankle sprain injury sustained during the team handball training. Twenty-six men and twenty-eight women completed the study (see Fig 1).

**Table 1:** Anthropometric characteristics of study participants.

Variable	THG	CON
Study participants	28	26
(m/w)	(14/14)	(12/14)
	(50%/50%)	(46%/54%)
Age (yrs)	24.1 ± 2.6	24.8 ± 3.1
Height (cm)	176.1 ± 8.6	176.0 ± 9.8
Weight (kg)	80.1 ± 16.6	78.4 ± 15.8
BMI	25.8 ± 4.6	25.2 ± 3.6
VO_2peak_ (ml-O_2_/kg/min)	39.8 ± 5.9	39.7 ± 5.0

Data represents means ± standard deviation. Number of study participants presented as total number and distribution into men (m) and women (w).

Fourteen men and fourteen women were randomized to small-sided team handball training (THG), while twelve men and fourteen women were randomized to no training (CON) (Table 1).

All participants were fully informed of the experimental procedures and possible discomfort associated with participation before giving their written informed consent. The study was carried out in accordance with the guidelines contained in the Declaration of Helsinki, after obtaining ethical approval by the National Committee on Health Research Ethics of Copenhagen (journal.nr. H-15008361). The present study was conceived and designed as a non-clinical randomized training intervention study in healthy young males and females. However, during the submission process the study was registered at ClinicalTrials.gov (ID number: NCT04247724) upon request from the PLoS ONE Editoral Office.

### Team handball training

Participants in THG were offered three supervised training sessions per week for 12 wks. The average attendance rate was 1.8±0.3 training sessions per week (range: 1.1–2.8). Training intensity has been reported in the referred papers from the main study [24, 25]. All THG training sessions were conducted at the University of Copenhagen using an indoor wooden floor team handball field (field size = 19 x 13.3 m) and consisted of a ~15-min warm-up program, followed by four 10-min bouts of small-sided team handball match-play, separated by 3-min rest periods. Warm-up consisted of low-to-moderate speed running, light strength training exercises and technical drills. The small-sided match-play consisted primarily of 4-vs-4 and 3-vs-3 matches according to training attendance, corresponding to 32–42 m^2^ per player. The balls used for the warm-up and games were soft rubber balls (Trial Butterfly Primo, Ø = 15 cm, weight = 150 g). Goal frames were 2 m wide and 1.7 m high and the arc line was 4.5 m from the middle of the goal line. During all match-play exercise, all teammates had to be on the opponent’s half-field before a goal could be scored. The scoring player subsequently always was assigned as goalkeeper in his/hers own goal, which ensured high-intensity return runs and made the players rotate positions in a random order. All training sessions were sex-specific, with men playing against men and women against women. Physical loading intensity during small-sided team handball match-play was measured by heart rate (HR) monitors (Polar Electro OY, Kempele, Finland). Mean HR for men and women was 83.8±3.8 and 84.9±6.3%HR_max_, respectively, with no differences between sexes (*p* = 0.59, independent samples t-test). HR_max_ was measured during an incremental treadmill test to exhaustion and determined as the highest value during a 15-s period. Measurement of training intensity was supplemented by time-motion analysis of selected match-plays to allow the analysis of selected intense action types such as jumps, turns and high-intensity runs (data published in [24, 25]).

### Control participants

Study participants allocated to CON were told to sustain their normal lifestyle without making any significant changes to their daily patterns of physical activity. After the 12 wks intervention including post testing, all control participants were invited to enrol in the handball training programme (with no test data obtained).

### Maximal muscle strength, power and postural balance

Dynamic maximal voluntary contraction (MVC) strength for the knee extensors was measured in an isokinetic dynamometer (KinCom, Kinetic Communicator 500-H, Chattecx Corp., Chattanooga, TN, USA) as previously described [12, 27]. In brief, force signals were sampled at 1,000 Hz using an analog-to-digital (A/D) converter (16-bit A/D converter, DT9804, Data translation, Malboro, MA, US) and subsequently digitally lowpass filtered using a fourth-order, zero-lag Butterworth filter with a cut off frequency of 15 Hz. During testing of maximal dynamic concentric strength, the range of knee joint motion (ROM) was from 90° flexion to 10° full extension, performed at a constant joint angular velocity of 60°/s. Study participants had five submaximal familiarization trials at ~50% preceding the MVC test. MVC was deemed to be reached, when study participant failed to increase peak moment two times in a row. Each trial was separated by one minute pause. Maximal voluntary isometric contraction strength (MVIC) of the knee extensor was obtained at 70° fixed knee joint angle (0° = full knee extension), during which rapid force capacity (contractile rate of force development: RFD) was assessed during the early (0–50 ms) and later (0–200 ms) phases of rising muscle force [20, 27]. Study participants performed two submaximal familiarization trials before performing three 4-s MIVC trials, with 1-minute pause between successive trials. MVC and MIVC measurements were always performed for the leg contralateral to the dominant hand, corresponding to the take-off leg when performing jump-shots in team handball. All torque signals were gravity corrected as described elsewhere [28].

As described in detail previously [14] postural balance was evaluated by measuring sway area and sway length for the center of pressure (CoP) during quiet 30-s unilateral (single leg, same as tested for MVC, MIVC and RFD) standing on a force plate (AMTI OR6-5-1000, Watertown, MA, USA). CoP was recorded at a sampling frequency of 100 Hz (16-bit A/D converter, DT9804, Data translation, Malboro, MA, US) using custom-made software (DueScope 1.3, 2012, Mathworks, Natrick, USA). Participants were instructed to stand with their hands on their hips and look straight forward with a 90-degree knee flexion in the freely hanging contra-lateral leg. Participants performed 3 trials separated by a 20-s rest period and the trial with the shortest CoP excursion length was chosen for further statistical analysis.

Maximal countermovement jumps (CMJ) were performed on a force plate (AMTI OR6-5-1000, Watertown, MA, USA) with vertical ground reaction force recorded at a 1000 Hz (16-bit A/D converter, DT9804, Data translation, Malboro, MA, USA). Maximal vertical jump height and maximal stretch-shortening cycle (SSC) leg extensor power were analysed as described in detail previously [14, 29, 30]. In brief three maximal jumps were performed separated by 20-s rest periods. Study participants were instructed to position their hands at their hips and to jump as high as possible. The jump with highest jump height was selected for further analysis.

### Body composition

Body fat percentage, whole-body fat free mass (FFM), regional leg FFM, bone mineral content (BMC), regional leg BMC, whole-body bone mineral density (BMD), local hip BMD, whole body T-score and local hip T-score were determined by dual X-ray absorptiometry (DXA) scans (LUNAR IDXA, GE Medical Systems, Madison, Wisconsin, US). The scans were performed on the same time of the day during pre and post testing, with participants fasting overnight (minimum 8 hours). Gender-specific data have been reported previously for these parameters [24, 25].

### Muscle biopsy sampling

Muscle biopsies (vastus lateralis) were obtained pre- and post-intervention for male participants only, as described in details elsewhere [31–33]. Briefly, muscle samples were mounted with Tissue-Tek (4583, Sakura Finetek Europe B. V., Alphen aan den Rijn, Holland) and immediately frozen in isopentane cooled with liquid nitrogen and stored at -80°C until later histochemical analysis. Serial sections (10μm) were cut in a cryostat (-20°C, Cyros ARNX70, Thermo Scientific, Walldorf, Germany) and ATPase staining with preincubation at pH 4.37, 4.54, and 10.30 were performed [31, 34]. In addition, immunohistochemistry staining of capillaries was performed using the double staining method [35].

Muscle fibres size and capillary density in muscle biopsies from 7 THG and 7 CON particiants were analysed, while muscle fibre type composition was determined in 11 participants from THG and in 9 from CON. Muscle biopsies from 6 participants had to be excluded from the analysis due to insufficient sample size or freeze damage to the tissue. The specific number of samples (participants) analysed for each parameter are specified in Table 4.

### Statistical analysis

The Shapiro-Wilk test was applied to test data for normal (Gaussian) distribution. Evaluation of pre-to-post between-group (time x group) differences (absolute units) was conducted using a two-way repeated-measures analysis of variance (two-way RM ANOVA). Dependent samples t-testing was performed to evaluate pre-to-post within-group changes (absolute units). Level of statistical significance was chosen as *p* ≤ 0.05. All statistical analysis was performed using IBM SPSS statistics v. 24, 2016.

## Results

### Muscle strength and postural balance

**Fig 2:**
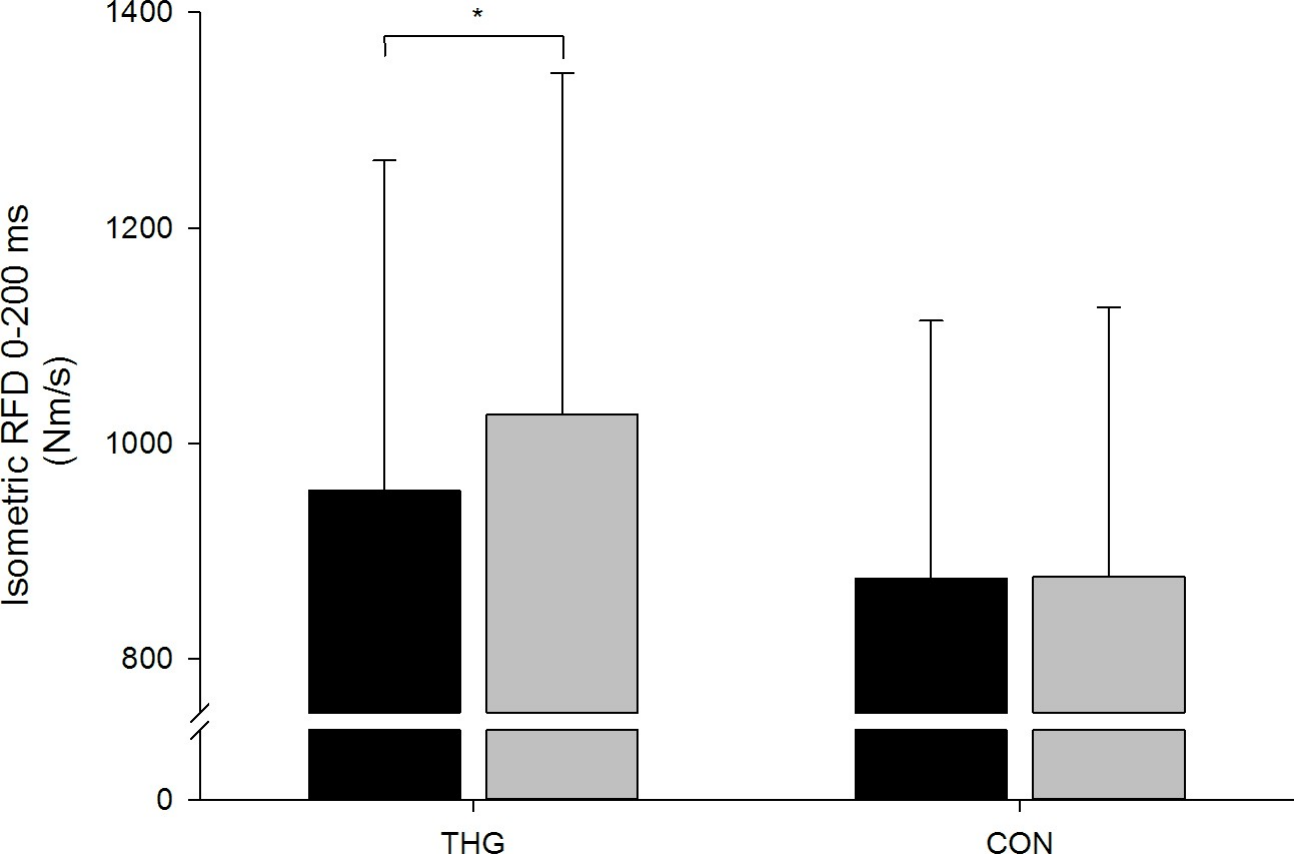
Late phase (0–200 ms) rate of force development (RFD). Late phase (0–200 ms) rate of force development (RFD) assessed during maximal isometric knee extensor strength (MVIC) testing at baseline (black columns) and following (grey columns) 12 wks small-sided team handball training (THG) or no training (CON). Data are presented as means ± standard deviation (SD). *Within-group different from baseline (p<0.05).

No systematic changes in dynamic (MVC) or isometric (MVIC) knee extensor strength were observed for neither THG nor CON after the intervention period. Further, no systematic change in early phase knee extensor RFD (0–50 ms) was observed. In contrast, late phase knee extensor RFD (0–200 ms) increased by 7.4% (*p* = 0.028) in THG while remaining unaltered in CON (+0.2%), with no between-group difference in this response (*p* = 0.172) (Fig 2 & Table 2).

**Table 2:** Muscle strength, CMJ height/power and postural balance.

	Baseline		12 wks		Change from baseline		Percentage change from baseline	T-distribution	df	With-in group	F-ratio	Time x group
Variable	Mean	SD	Mean	SD	Mean	SD				*p*-value		*p*-value
Isokinetic MVC (Nm)												
THG n = 24 (10)	230.1	70.2	239.3	69.5	9.2	26.1	4.0%	-1.736	23	0.096	0.442	0.509
CON n = 26 (14)	213.3	56.1	218.1	54.4	4.8	21.0	2.3%	-1.164	25	0.255		
Isometric MVC (Nm)												
THG n = 25 (11)	270.3	75.7	275.4	72.5	5.1	25.7	1.9%	-0.980	24	0.337	0.444	0.508
CON n = 26 (14)	253.2	70.8	262.9	74.4	9.7	24.9	3.8%	-1.994	25	0.570		
Isometric RFD_0-50 ms_ (Nm/s)												
THG n = 24 (11)	1397.4	709.1	1400.4	658.1	3.0	590.3	0.2%	-0.513	24	0.981	0.174	0.679
CON n = 23 (12)	1110.7	499.5	1191.5	658.6	80.8	687.2	7.3%	-0.836	25	0.579		
Isometric RFD_0-200 ms_ (Nm/s)												
THG n = 24 (11)	956.0	306.5	1026.6	316.9	70.6	147.8	7.4%	-2.575	24	0.028*	1.930	0.172
CON n = 23 (12)	874.3	239.6	876.2	250.0	1.9	189.5	0.2%	-1.233	25	0.963		
Sway area (cm^2^)												
THG n = 27 (13)	686	181	637	161	-49	162	-7.1%	1.575	26	0.127	1.634	0.207
CON n = 26 (14)	701	229	727	325	26	257	3.7%	-0.519	25	0.609		
Sway length (mm)												
THG n = 27 (13)	937	268	853	198	-84	217	-9.0%	2.015	26	0.054*	1.357	0.249
CON n = 26 (14)	930	311	933	369	3	321	0.3%	-0.051	25	0.960		
CMJ jump height (cm)												
THG n = 27 (13)	24.8	7.8	25.4	7.4	0.6	2.1	2.4%	-1.494	26	0.147	0.242	0.625
CON n = 26 (14)	21.8	6.0	22.1	6.3	0.3	2.0	1.5%	-0.847	25	0.405		
CMJ P_peak_ (W/kg)												
THG n = 27 (13)	42.6	8.3	43.3	8.3	0.7	2.6	1.5%	-1.284	26	0.210	0.167	0.685
CON n = 26 (14)	38.8	6.9	39.9	7.4	1.1	5.1	2.9%	-1.118	25	0.274		

*Different from baseline (*p*≤0.05). Means ± standard deviation for both the team handball group (THG) and control group (CON) before (baseline) and after (12 wks) the intervention period. The mean change score for group and standard deviation (SD) (change from baseline) is presented along with the percentage change from baseline. Table 2 shows the within-group T-distribution degrees of freedom (df) and *p*-values, along with F-ratio and between-group *p*-values. Strength parameters isokinetic and isometric maximal voluntary contraction force, early and late phase rate of force development (RFD 0–50 ms, RFD 0–200 ms, respectively) are presented. The variables of postural balance sway area and sway length along with the jump height and peak power from the countermovement jump test (CMJ) are also presented.

**Fig 3:**
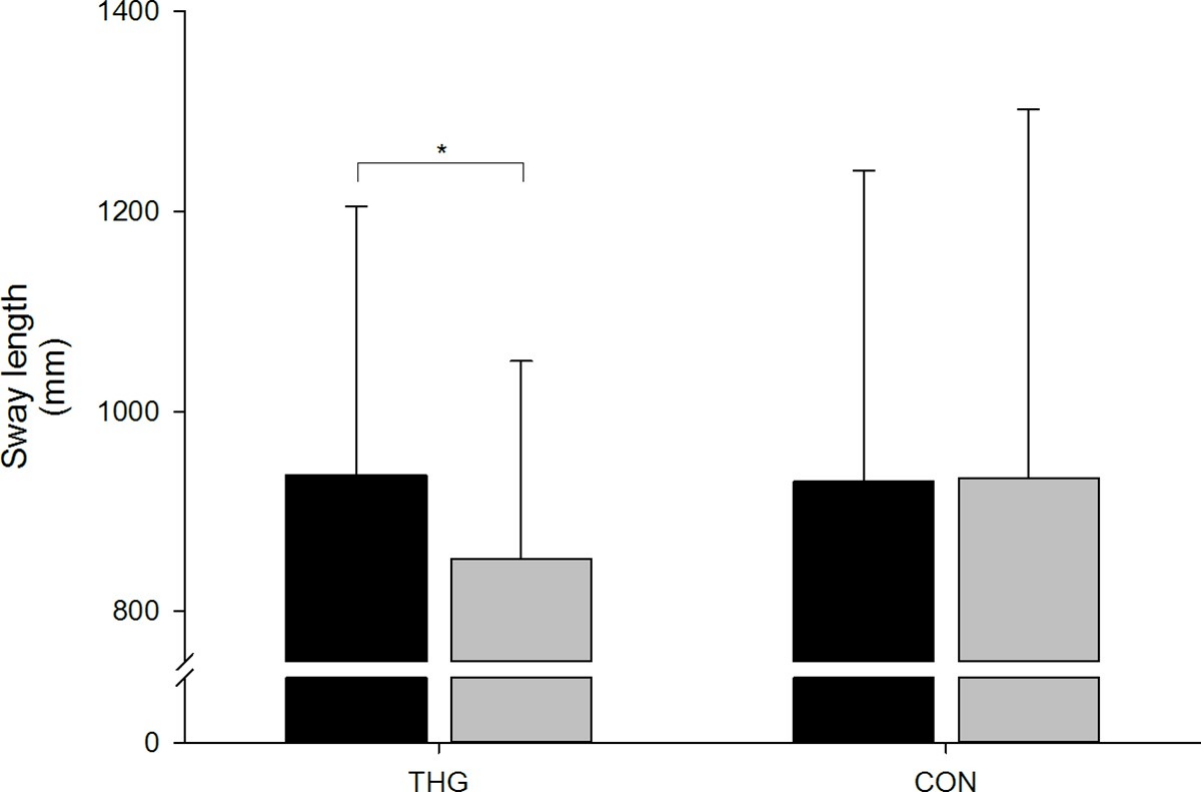
Postural balance. Postural balance assessed as center of pressure (CoP) sway length during 30-sek unilateral static balance test at baseline (black columns) and following (grey columns) 12 wks small-sided team handball training (THG) or no training (CON). Mean ± standard deviation (SD) are presented. *Within-group difference compared to baseline (p<0.05).

CoP sway length was reduced in THG (-9.0%, *p* = 0.054), whereas there was no change in CON and no significant between-group difference (Fig 3 & Table 2).

No changes in CoP sway area or CMJ performance (maximal vertical jump height or take-off power) were observed in THG or CON (Table 2).

### Body composition

After the 12 wks training intervention period, there was a time-by-group interaction effect (*p* = 0.029) in body fat percentage, with THG demonstrating a 3.7% decrease (*p*<0.001), whereas no change was observed in CON (-0.8%, *p* = 0.467) (Table 3). In addition, FFM increased by 2.2% in THG (*p*<0.001) and 1.0% in CON (*p* = 0.018), with THG increasing more (*p* = 0.037) than CON (Table 3). Regional leg FFM increased 2.5% for THG (*p*<0.001) and 1.3% for CON (*p* = 0.015) with similar magnitude of gains (*p* = 0.151) in THG and CON (Table 3).

**Table 3:** Body composition.

	Baseline		12 wks		Change from baseline		Percentage change from baseline	T-distribution	df	With-in group	F-ratio	Time x group
Variable	Mean	SD	Mean	SD	Mean	SD				*p*-value		*p*-value
Body fat percentage (%)												
THG n = 28 (14)	32.7	8.7	31.5	8.6	-1.2	1.5	-3.7%	4.137	27	<0.001*	5.044	0.029*
CON n = 26 (14)	31.7	7.9	31.4	8.1	-0.3	1.6	-0.9%	0.739	25	0.467		
FFM (kg)												
THG n = 28 (14)	53.2	9.2	54.3	9.5	1.2	1.1	2.2%	-5.816	27	<0.001*	4.564	<0.037*
CON n = 26 (14)	53.3	11.5	53.8	12.0	0.5	1.1	1.0%	-2.530	25	0.018*		
FFM legs (kg)												
THG n = 28 (14)	19.2	3.8	19.7	4.0	0.5	0.6	2.5%	-4.657	27	<0.001*	2.574	0.151
CON n = 26 (14)	18.9	4.4	19.2	4.5	0.3	0.5	1.3%	-2.617	25	0.015*		
BMC (kg)												
THG n = 28 (14)	2.84	0.41	2.87	0.42	0.03	0.05	1.1%	-3.405	27	0.002*	7.151	0.010*
CON n = 26 (14)	2.83	0.57	2.83	0.56	0.00	0.03	0.0%	-0.180	25	0.859		
BMC legs (kg)												
THG n = 28 (14)	1.05	0.20	1.06	0.20	0.01	0.02	1.2%	-4.234	27	<0.001*	9.175	<0.004*
CON n = 26 (14)	1.05	0.25	1.05	0.24	0.00	0.02	-0.4%	0.880	25	0.387		
Total body BMD (g/cm^2^)												
THG n = 28 (14)	1.265	0.096	1.268	0.091	0.003	0.022	0.2%	-0.733	27	0.470	0.329	0.569
CON n = 25 (13)	1.251	0.129	1.250	0.126	-0.001	0.018	-0.1%	-0.043	24	0.966		
Total hip BMD (g/cm^2^)												
THG n = 28 (14)	1.099	0.115	1.117	0.115	0.018	0.017	1.6%	-5.410	27	<0.001*	21.844	<0.001*
CON n = 25 (13)	1.061	0.137	1.060	0.140	-0.001	0.011	-0.1%	0.590	24	0.561		
Total body T-score												
THG n = 28 (14)	1.2	0.9	1.3	0.9	0.1	0.2	8.3%	-1.007	27	0.323	0.284	0.596
CON n = 25 (13)	1.1	1.2	1.1	1.2	0.0	0.2	0.0%	-0.312	24	0.758		
Total hip T-score												
THG n = 28 (14)	0.4	0.9	0.6	0.9	0.2	0.1	50.0%	-5.014	27	<0.001*	18.266	<0.001*
CON n = 25 (13)	0.2	1.1	0.1	1.1	-0.1	0.1	-50.0%	0.749	24	0.461		

*Different from baseline (*p*≤0.05). Means ± standard deviation for both the team handball group (THG) and control group (CON) before (baseline) and after (12 wks) the intervention period. The mean change score for group and standard deviation (SD) (change from baseline) is presented along with the percentage change from baseline. Table 3 shows the within-group T-distribution, degrees of freedom (df) and *p*-values, along with F-ratio and between-group *p*-values. Body fat percentage (%), free fat mass (FFM (g)), bone mineral content (BMC (g)), bone mineral density (BMD (g/cm^2^)) and T-score (total body T-score) for the whole body are shown in the table. Furthermore, fat free mass (FFM legs (g)) and bone mineral content (BMC (g)) for the legs are presented. In addition, T-score for the whole body (total body T-score) and hip (total hip T-score) are presented.

### BMC and BMD

Total BMC and regional leg BMC increased 1.1–1.2% (*p*<0.05) with training in THG, with both parameters changing more (*p* = 0.010) than in CON (Table 3). There was no change in total body BMD. THG showed an increase in hip BMD (+1.6%, *p*<0.001), which was different from CON (*p*<0.001) (Table 3). No change was observed for total body T-score, but an increase in total hip T-score (+50%, *p*<0.001) was observed in THG following training which exceeded the change in CON (*p*<0.001) (Table 3).

### Muscle fibre composition and morphology

No systematic changes in specific fibre type CSA or fibre type composition were observed at post-training (Table 4). No pre-to-post changes in capillarization were observed (*p*>0.05) (Table 4). The relative (%) magnitude of between-group changes did not differ for any of the muscle fibre parameters obtained.

**Table 4:** Muscle fibre (VL) and capillary analysis.

	Baseline		12 wks		Change from baseline		Percentage change from baseline	T-distribution	df	With-in group	F-ratio	Time x group
Variable	Mean	SD	Mean	SD	Mean	SD				*p*-value		*p*-value
**Fibre type distribution**												
Type I (%)												
THG n = 11 (0)	47.0	14.8	39.2	14.6	-7.8	16.9	-16.6%	1.531	10	0.157	0.033	0.859
CON n = 9 (0)	45.7	14.4	39.1	13.1	-6.6	11.5	-14.4%	1.722	8	0.123		
Type IIa (%)												
THG n = 11 (0)	37.6	11.0	44.2	9.3	6.6	13.0	17.6%	-1.689	10	0.122	0.629	0.438
CON n = 9 (0)	34.6	7.2	37.2	11.3	2.6	8.2	7.7%	-0.964	8	0.363		
Type IIx (%)												
THG n = 11 (0)	15.5	9.9	16.6	10.7	1.1	11.7	7.4%	-0.325	10	0.752	0.216	0.648
CON n = 9 (0)	19.8	13.1	23.7	9.1	3.9	15.1	19.9%	-0.779	8	0.458		
**Fibre type cross sectional area**												
Type I (μm^2^)												
THG n = 7 (0)	5246	1055	6572	2004	1326	1756	25.3%	-1.998	6	0.093	1.573	0.234
CON n = 7 (0)	5125	1568	5207	644	82	1951	1.6%	-0.111	0	0.915		
Type IIa (μm^2^)												
THG n = 7 (0)	7064	1300	7877	3216	814	3145	11.5%	-0.684	6	0.519	0.362	0.559
CON n = 7 (0)	6682	2627	6653	1163	-30	1965	-0.4%	0.040	6	0.970		
Type IIx (μm^2^)												
THG n = 7 (0)	6640	1902	5859	2620	-781	3595	-11.8%	0.575	6	0.586	0.239	0.634
CON n = 7 (0)	5283	2326	5222	1767	-61	1511	-1.2%	0.107	6	0.918		
**Fibre area percentage distribution**												
Type I (%)												
THG n = 7 (0)	38.8	15.6	38.8	11.4	0.0	14.9	0.0%	-0.003	6	0.998	0.037	0.851
CON n = 7 (0)	35.4	13.0	36.9	16.2	1.6	15.7	4.5%	-0.266	6	0.799		
Type IIa (%)												
THG n = 7 (0)	45.5	15.4	50.2	9.0	4.7	15.4	10.4%	-0.813	6	0.447	0.290	0.600
CON n = 7 (0)	43.5	11.1	44.2	10.5	0.7	12.6	1.6%	-0.144	6	0.890		
Type IIx (%)												
THG n = 7 (0)	15.7	12.5	11.0	8.5	-4.7	9.0	-30.2%	1.393	6	0.213	0.202	0.661
CON n = 7 (0)	21.2	12.5	18.9	9.4	-2.3	11.0	-11.0%	0.560	6	0.596		
**Capillaries**												
Cap./fibre												
THG n = 7 (0)	2.1	0.3	2.2	0.6	0.1	0.8	4.8%	-0.444	6	0.673	0.147	0.708
CON n = 7 (0)	1.9	0.5	1.9	0.2	0.0	0.5	-0.4%	0.036	6	0.973		
Cap./mm^2^												
THG n = 7 (0)	346.4	37.4	342.3	127.9	-4.1	127.0	-1.2%	0.086	6	0.934	0.044	0.838
CON n = 7 (0)	355.6	90.4	339.9	50.1	-15.7	72.3	-4.4%	0.575	6	0.586		

*Different from baseline (*p*≤0.05). Data reflects analysis of muscle biopsies from the men in both the team handball group (THG) and control group (CON) before (baseline) and after (12 wks) the intervention period. Means ± standard deviation at baseline and after 12 wks intervention is presented. Mean change score and standard deviation for each group (change from baseline) and percentage change from baseline is also presented. Table 4 shows furthermore the within-group T-distribution degrees of freedom (df) and *p*-values, along with F-ratio and between-group *p*-values.

## Discussion

The present study investigated the effects of recreational small-sided team handball training on lower limb muscle strength, myofiber morphology, fat free mass, bone health and postural balance in young untrained men and women. The main findings were that team handball training led to increases in rapid force capacity (increased late phase RFD) and postural balance (diminished CoP sway excursion). Equally important, team handball training elicited significant gains in whole-body and lower limb fat free mass, decreased body fat percentage along with increases in whole-body BMC, regional leg BMC and hip BMD, respectively.

No increase in dynamic or static muscle strength or power was observed following training in this study. Expectations of an enhanced lower limb muscle strength was based on previous findings in the comparable intermittent sport; football fitness. An explanation for the missing strength improvements in m. quadriceps could be that more training time is needed. A study of Krustrup and colleagues (2010) showed that 16 months of small-sided football fitness could elicit improvements in both dynamic and static muscle strength of m. quadriceps, with no difference between 0 to 4 moths [36].

Study participants demonstrated a substantial increase (7.4%) in rapid force capacity, assessed as late phase (0–200 ms) RFD following 12 wks of small-sided team handball training (Fig 2). No previous studies have investigated the effect of small-sided team handball training on rapid force capacity (RFD) [24–26, 37, 38], whereas a few reports exist on the effects of small-sided recreational football training on the adaptive plasticity of lower limb RFD. Thus, when examined in older men (68 yrs), knee flexor (m. hamstring) RFD increased by 89% following 12-months of small-sided football fitness training (1.7 weekly training sessions), whereas knee extensor RFD remained unaffected [12]. Further, small-sided football fitness training for 16 months (1.9 sessions per week) in young to middle-aged women (19–47 yrs) resulted in marked improvements (+35–65%) in peak quadriceps RFD and contractile impulse, accompanied by comparable gains (+50%) in peak hamstring RFD [36]. These observations indicate that long-term participation in small-sided recreational ball games can increase lower limb rapid force capacity in both young and elderly men and women. The present data add to these findings by demonstrating that short-term (12 wks) effects of recreational small-sided game activities on lower limb RFD may also be achieved for young adults.

It has been previously established that resistance training is highly effective for increasing rapid force capacity (RFD) using short-term interventions (12–16 wks) in young, as well as elderly individuals [27, 39, 40]. The present protocol of small-sided team handball training showed increases in knee extensor RFD, that were somewhat lower in relative magnitude (~10% vs. 20–35%) compared to the gains in RFD typically reported with short-term isolated resistance training (cf. above studies). A number of physiological factors are known to affect rapid force capacity: myofibre CSA, efferent neural drive to myofibres and myofibre type composition, to mention just a few [41, 42]. Early phase RFD is mainly determined by intrinsic muscle contractile properties [43] and the magnitude of efferent neural drive (maximal muscle unit discharge rates) [44, 45], whereas late phase RFD has been positively associated with muscle mass and maximal voluntary muscle force [43]. The strong association between late phase RFD and maximal force generating capacity of the muscle (MVC) [43] might explain the present increase in late phase knee extensor RFD, since a 4% increase (not significant *p*>0.05) quadriceps MVC was observed for THG in the present study. Further, the increased regional leg FFM observed in THG probably also has contributed to the post-training increase in late phase RFD. Altogether, these observations suggest that muscle mass and/or perhaps also muscle activation, can be stimulated through short-term small-sided team handball training in young previously untrained men and women.

Furthermore, an association between reduced RFD and impaired postural balance (discussed separately below), potentially implying an increased risk of falls, has been reported in both old [46] and young adults [14]. The foundation for a high rapid force capacity (RFD) is likely formed already in early adulthood, and from the present data it seems that short-term participation in small-sided team handball training may be an effective tool to achieve positive adaptations in RFD in young adults.

In the present study, postural balance was improved after 12 wks of small-sided team handball training as manifested by a reduced CoP sway excursion (Fig 3). Postural balance reflects the ability to control centre of mass (CoM) in relation to base of support (BoS) within the limits of stability (LoS). A previously reported negative correlation between early phase (0–50 ms) knee extensor RFD and postural balance (CoP sway area) in young men (25–35 yrs) [14], indicates that a fast muscle activation in the initial phase of the muscle contraction is essential for optimal postural balance. Interestingly, a negative relationship was observed between the percentage of type I muscle fibre area and CoP sway area, suggesting that postural balance is also dependent on slow-twitch type I muscle fibre function. In the present study, no longitudinal changes were observed in early phase RFD or in the percentage area of type I muscle fibres, nor did we observe any relationship between these parameters at baseline. The reduced CoP sway excursion observed post-training in the present study for THG is likely the combined result of elevated lower limb RFD and/or altered patterns of neuromuscular activation. Previously, comparison of postural balance with concurrent surface electromyography (SEMG) measurements between young adults (23.3 yrs) and seniors (62.7 yrs) showed a shorter CoP sway excursion and a higher muscle activation (elevated SEMG) in young subjects [47].

As suggested by Jakobsen and colleagues (2011), the high rate of directional footstep changes performed during small-sided football fitness training may have been the main reason for their observed improvements in postural balance. In support of this notion, reductions were also noted in medio-lateral CoP sway variability, which were suggested to result from the large number of lateral side-cuts and turns performed during training [14]. Likewise, small-sided team handball is characterized by a high-frequency of anaerobic short-lasting actions [24–26] that includes rapid changes in direction such as those implied in side-cutting manoeuvres, which may therefore, constitute another possible explanation for the improved postural balance observed in the present study. Interestingly, when static unilateral postural balance (CoP velocity) was compared between sub-elite and elite football players (18–30 yrs), elite players demonstrated superior postural balance [48]. Thus, exercise induced improvements in postural balance might be dependent on the total amount of training and/or the level of technical performance when exercising. Regardless the specific mechanisms involved, the present study clearly demonstrates that short-term, low-frequency small-sided team handball training has a positive effect on postural balance in young adults. Speculatively, this specific type of training might have an even greater positive impact on postural balance in elderly individuals, which may serve to reduce the risk of falls in this population, a subject to be addressed in future studies.

The present study demonstrated increases in whole-body BMC and regional leg BMC following THG training. In addition, longitudinal changes in local hip BMD and hip T-score were greater in THG than in CON. These gains in bone biomarkers support recent findings of sex-specific improvements in bone health following 12 wks of small-sided team handball training for young adults [24, 25]. A high magnitude of strain on the skeleton is required to induce a maximal exercise-osteogenic response, which can be achieved by performing rapid and forceful changes in direction when running [11]. The intermittent and space-restricted nature of the small-sided team handball game format results in a high-frequency of jumps, turns, side-cutting and changes of direction [24–26], which is likely to result in a high impact and strain on the bones exposed to exercise. Small-sided football fitness is a comparable intermittent game type which has previously been suggested to elicit substantial improvements in bone structure for young, middle-aged and elderly participants [11, 49]. With 12–16 wks of 1–3 hours weekly, small-sided football fitness training improved clinically important sites of bone structure, such as the femoral shaft and trochanter, with 1–3% gains in BMD in young to middle-aged untrained men, hypertensive women and elderly men, as well as in prostate cancer patients [18, 49, 50]. The study of Uth et al., (2016) revealed a significant correlation between the number of decelerations performed during training and the training-induced changes in leg BMC (r = 0.65, *p* = 0.012) and between the total number of accelerations and the changes in leg BMC (r = 0.59, *p*<0.05). These observations support the notion that the use of intense intermittent game formats and a small number of players on the playing field leads to improved bone health, probably due to the high-impact strain stimulation imposed on the skeleton. Similar mechanisms are likely to play a role in small-sided team handball training, given the intermittent exercise nature with many accelerations, decelerations and highly intense muscle actions [24, 25].

### Limitations

Study participants were instructed not to change their habitual activity pattern during the 12-wks intervention period. All participants filled out the International Physical Activity Questionnaire (IPAQ) [51] before and after the intervention period, which showed that, except from the small-sided team handball training in HTG, none of the study participants had changed their activity pattern [24, 25]. Nevertheless a discrepancy may exist between self-reported physical activity and physical activity measured by accelerometer [52, 53]. Thus, future studies should monitor the magnitude of non-prescribed physical activity during comparable intervention regimes. For statistical analyses it would have been preferential to use a generalized linear mixed model (GLMM), but due to loss of statistical power (<0.80) the authors decided to apply a two-way RM ANOVA. Only muscle samples from male study participants were collected. Due to insufficient sample size or freeze damage to the tissue a very small sample size was obtained. Therefore, interpretation of the muscle fibre analysis should be done with caution as results are sensitive to a very small change in a few participants.

## Conclusions

The present study investigated the physical health and fitness benefits of short-term (12 wks) recreational team handball training in young adults. In contrast to our initial hypothesis, 12 wks team handball training appeared to have no effect on lower limb muscle strength in the present group of untrained adults, since no improvements in dynamic or static knee extensor muscle strength were observed. Nonetheless, substantial gains in rapid force capacity (RFD) and postural balance were observed following the training period, accompanied by increases in lean body mass and bone mineralization and decrease in fat body mass. These observations add to previous reports in untrained adults of positive effects of small-sided team handball training on cardiovascular, metabolic and musculoskeletal fitness, respectively [24–26, 37].

The potential stimuli for the positive adaptations emerging in the present study could be related to the high number of intense muscle actions (accelerations and decelerations, changes of direction, side-cutting manoeuvres) inherent to small-sided team handball practice. In conclusion, small-sided team handball is a physical activity that appears to effectively improve musculoskeletal fitness. It is an activity that has simple demands and can be performed without any preunderstanding, for which reasons it may be implemented to improve musculoskeletal fitness and enhance physical health.

## Supporting information

S1 Fig (PDF)

S1 Data (XLSX)

S2 Data (XLSX)

S1 File (XLSX)

S2 File (PDF)

S3 File (PDF)

S4 File (PDF)
