# Supplementary figures and images for: Effects of small-sided recreational team handball training on mechanical muscle function, body composition and bone mineralization in untrained young adults—A randomized controlled trial
Source: PLoS One. 2020 Nov 18;15(11):e0241359. doi: 10.1371/journal.pone.0241359 (PMC7673568; doi:10.1371/journal.pone.0241359)

Figure 1

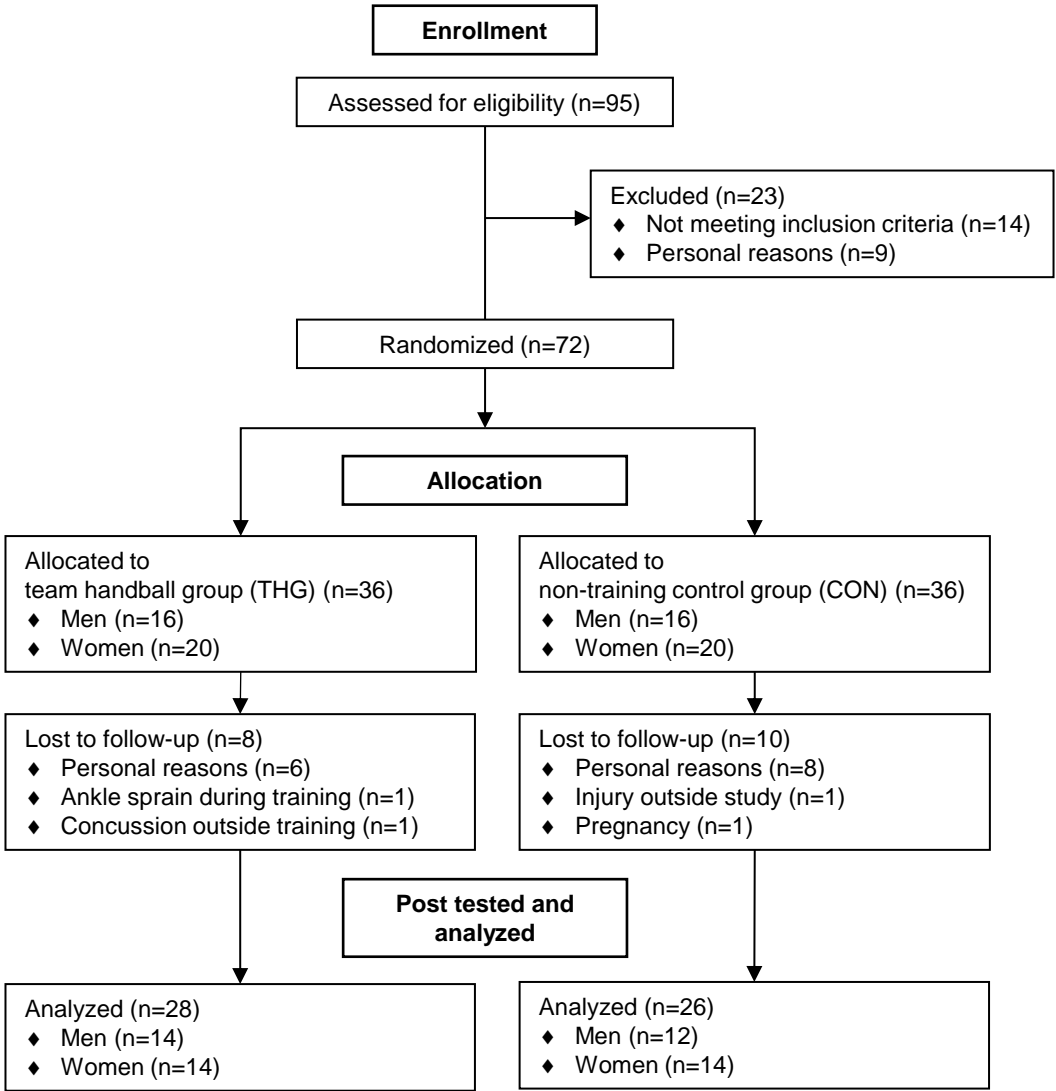

Supplement: S1 Fig — (PDF) [file pone.0241359.s001.pdf]
