# Supplementary material for: Effects of small-sided recreational team handball training on mechanical muscle function, body composition and bone mineralization in untrained young adults—A randomized controlled trial
Source: PLoS One. 2020 Nov 18;15(11):e0241359. doi: 10.1371/journal.pone.0241359 (PMC7673568; doi:10.1371/journal.pone.0241359)
Supplement: S3 File — (PDF) [file pone.0241359.s006.pdf]

Journalnummer: H-15008361

Hovedprotokol_v2_29-06-2015

Anmeldelse til Videnskabsetisk Komité,

De Videnskabsetiske Komitéer for Region Hovedstaden

Regionsgården, Kongens Vænge 2, 3400 Hillerød

Forskningsprojekt om motionshåndbold og sundhed

Baggrund

Der er en stor forekomst af livsstilssygdomme i det moderne samfund, såsom hjertekar-sygdomme, type 2 sukkersyge og knogleskørhed. Fysisk inaktivitet er en af de største risikofaktorer for udvikling af livsstilssygdomme. Nyere forskning har vist at motionsfodbold virker som en effektiv og bredspektret forebyggelse og behandling af livsstilssygdomme pga. positive effekter på kardiovaskulær, metabolsk og muskuloskeletal fitness (Krustrup 2010, 2013; Uth et al. 2014; Bangsbo et al. 2015). Det gælder utrænede mænd og kvinder på 20, 30, 50 og 70 år og patientgrupper med forhøjet blodtryk, type 2 sukkersyge og prostatakræft. Samtidig er det observeret, at motionsfodbold opbygger venskaber og skaber sociale relationer, som er vigtige elementer for at optimere deltagelse og fastholdelse af fysisk aktivitet (Nielsen et al. 2014; Ottesen et al. 2010). Motionsfodbold er blevet anerkendt som en sundhedsfremmende holdspilsidræt, der kan forbedre både inaktive raske og syge gruppers livskvalitet (Oja et al. 2015). Tesen er, at motionshåndbold har mange af de samme sundhedsmæssige kvaliteter som motionsfodbold pga. en intens interval træningsform med mange retningsskift og høj impact. Et pilotstudie på mænd (+40) med tidligere håndbolderfaring er allerede blevet gennemført i Portugal. Studiet viste, at deltagerne havde en høj intensitet under træning som er set i motionsfodbold for samme aldersgruppe. Endvidere havde deltagerne positive effekter på kondital og kropskomposition efter træningsperioden (Póvoas et al. 2012). Set i lyset af de mange sundhedsfremmende og sociale elementer som motionsfodbold besidder, vil det være

spændende at undersøge motionshåndbold nærmere i forhold til at finde flere boldspil, der kan bidrage til en sundere levevis blandt folk, der er inaktive eller ikke dyrker regelmæssig fysisk aktivitet.

Formål

Formålet er herved at undersøge fitness-og sundhedseffekter ved deltagelse i motionshåndbold i 12 uger for utrænede mænd og kvinder i alderen 18-30 år.

Forsøgets design og metoder

32 raske utrænede mænd (Kondital: 30-45 ml O2 min-1 kg-1) og 32 raske utrænede kvinder (Kondital: 25-40 ml O2 min-1 kg-) i alderen 18-30 år rekrutteres til at gennemføre 12 uger i enten a) en gruppe der spiller motionshåndbold eller b) en kontrolgruppe, der forsætter deres normale livsstil. Studiet er et randomiseret kontrolleret design. Forsøgspersonerne randomiseres efter stratificering for VO2max og fedtprocent til de 2 ovennævnte grupper, der gennemfører 12 ugers træning eller fortsættelse af inaktiv livsstil. De mandlige og kvindelige forsøgspersoner skal ikke indgå i samme træningsgruppe eller kontrolgruppe. Derfor vil der være 2 træningsgrupper (en mandlig og en kvindelig) og 2 kontrolgrupper (en mandlig og en kvindelig) med 16 forsøgspersoner i hver gruppe.

Træningsprotokol

Træningen vil bestå af 2-3 ugentlige træningspas af ca. 70 minutters varighed i 12 uger. De første 15 min er afsat til opvarmning, hvorefter der spilles 4*12 [illegible]nutter med 3 minutters pause mellem hvert spil. Den mandlige og kvindelige kontrolgruppe fortsætter med uændret livsstil. Træningssessionerne vil finde sted på Institut for Idræt og Ernæring, Nørre Alle 53, København N.

Tests

Der gennemføres testning før træningsperioden (1. testrunde) og efter 12 ugers træning (2. testningsrunde). Disse testdage har til formål at undersøge forsøgspersonernes kardiovaskulære, metabolske og muskuloskeletale fitness samt kortlægge en række betydningsfulde sundhedsparametre, herunder blodtryk, karfunktion, blodsukker, kolesterol, fedtprocent og knoglemineralisering. Følgende beskrives de 4 testdage, som foregår med minimum 48 timers mellemrum.

Testdag 1

Testdag 1 finder sted på Institut for Idræt og Ernæring, Universitetsparken 13, 2100 København Ø. Testdagen startes med en blodtryksmåling for at støtte op om at forsøgspersonen er sund og rask. Forsøgspersonen har til en indledende samtale, der ligger på en dag før første testdag, fået grønt lys om at deltage i projektet ud fra de oplysninger, som er givet til det veluddannede personale (Professor, Peter Krustrup, Human Fysiolog og PhD studerende, Therese Hornstrup, Human Fysiolog). Blodtryksmålingen efterfølges af en løbebåndstest, som består af 2 gange 5 minutters løb ved 6,5 og 8 km/t, efterfulgt af en maksimaltest, hvor der løbes i 4 minutter ved 8 km/t, hvorefter løbehastigheden øges med 1 km/t hvert minut til udmattelse. Åndedræt og sammensætningen af udåndingsluft måles, hvorved konditallet kan bestemmes. Testdag 1 varer 1,5 time.

Testdag 2

Testdag 2 finder sted både på Frederiksberg og Østerbro afdelingerne af Institut for Idræt og Ernæring. Dagen starter med at der udtages en fasteblodprøve fra en armvene med henblik på måling af blodsukker, langtidsblodsukker, insulin, hæmatokritværdi, kolesterol, inflammationsmarkører og knoglemarkører (~40 ml blod). Dette følges op af en spørgeskemaundersøgelse, som deltagerne udfylder på en computer. Spørgeskemaundersøgelsen består af spørgsmål til socioøkonomisk status, aktivitetsniveau (International Physical Activity Questionnaire: Craig et al. 2003), psykisk helbred (Hospital Anxiety Depression Scale: Zigmond & Snaith, 1983) velvære (Bradley 1994) og motivation til idræt generelt (Sport Motivation Scale:

Pelletier et al. 1995). Alle disse spørgeskemaer er validerede i dansk form eller i den engelske form, de er oversat fra, og passer til målgruppen. Dernæst gennemføres en helkrops DXA-scanning til bestemmelse af fedtprocent, muskelmasse og knoglemasse samt knogletæthed. Efter 30 minutters hvile foretages der 5 på hinanden følgende blodtryksmålinger efterfulgt af en endothelfunktionstest, som er en non-invasiv metode til at måle karfunktionen. Karfunktionen måles i liggende hvile ved hjælp af p*erifer arterietonometri* (PAT) metoden. PAT måler pulsbølgeamplituden under reaktiv hyperæmi ved hjælp af en fingerpletysmograf. Til sidst udtages en muskelprøve fra lårmuskulaturen under bedøvelse. Denne benyttes til histokemisk bestemmelse af fibertypefordeling og fibertypespecifik kapillarisering, enzymatiske analyser og metabolske analyser. Testdag 2 varer 3 timer.

Testdag 3

Testdag 3 finder sted på Institut for Idræt og Ernæring, Universitetsparken 13, København Ø og Bispebjerg Hospital, Bygning 10 stuen, 2400 København NV. *Ekkokardiografi:* Hjertefunktionen måles ved avanceret vævsdoppler ekkokardiografi til undersøgelse af muskelvævets kontraktions- og relaxationsmønstre. Der anvendes 2-D strain analyser, som tillader vinkel uafhængige analyser af såvel longitudinelle som radielle kontraktions mønstre og i tillæg vurderes circumferentielle bevægemønstre, og dermed selve twist bidraget til den globale myokardiefunktion. Undersøgelser foretages af sammenhæng mellem radial og longitudinel kontraktion, relation mellem twist (circumferentiel rotation ved apex og basis, relation mellem twist og diastolisk funktion, relation mellem twist, diastolisk funktion og atriedimension og ændringer under belastning. Målingerne foregår mens forsøgspersonen er i liggende position på siden. *Muskelfunktion:* Efter en standardiseret opvarmning på ergometercykel foretages en Nottingham power rig test til måling af maximal benmuskelpower, postural sway balancetest på et ben (henholdsvis statisk og med frontalt indhop) og til sidst måling af maximal dynamisk (koncentrisk) og isometrisk (statisk) muskelstyrke for knæextensor muskulaturen (dominante ben) i et isokinetisk dynamometer, (KinCom) inkl. måling af maximal explosiv muskelstyrke (Rate of Force Development: RFD).

Testdag 4

Testdag 4 finder sted i forbindelse med første træningssession på Institut for Idræt og Ernæring, Nørre Allé 53, København N. Der gennemføres en Yo-Yo Interval Udholdenhedstest – niveau 1. I forbindelse med denne test løbes 2x20 meter frem og tilbage mellem toppemarkeringer. Efter hver tur holdes 5 sekunders pause. Hastigheden under løbeturene justeres gradvist opad efter bip-signaler på en CD. Når man første gang ikke når tilbage til målstregen i tide, får man en advarsel, og anden gang er testen slut. Den tilbagelagte distance noteres som testresultat.

Ud over de førnævnte testdage vil der i forbindelse med udvalgte træningssessioner, udleveres et pulsbælte og en GPS-måler til måling af intensitet og arbejdsform under træningen. På de udvalgte træningssessioner skal der ydermere svares på et spørgeskema om hårdhed af træningssessionen, ømhed efter træning, oplevelsen af flow under træningen (Flow Kurz Skala: Rheinberg et al. 2003), oplevelsen af nydelse af træningen (Physical Activity Enjoyment Scale: Kendzierski & DeCarlo, 1991), og motivation til håndbold (Sport Motivation Scale: Pelletier et al.1995).

For de deltagere der endvidere har lyst, vil der være mulighed for at afgive en fæces- og urinprøve før og efter interventionen til senere at kunne karakterisere tarmbakteriesammensætning og tarmens funktionelle potentiale. Prøverne opsamles i forsøgspersonernes eget hjem, og de får det nødvendige udstyr til at kunne udføre procedure samt en grundig gennemgang af hvordan prøverne skal håndteres i forhold til opbevaring og hygiejne. Prøverne fryses ved -18 grader og personale fra Københavns Universitet vil inden for en uge hente prøverne og transportere dem til Universitet for yderligere opbevaring. Alle prøverne samles i følge standardprocedure til efterfølgende standardiseret DNA og RNA ekstraktion.

Forsøgspersonerne, herunder inklusions- og eksklusionskriterier

Forsøgspersonerne skal være raske men utrænede, mænd: kondital 30-45 ml O2 min-1 kg-1, kvinder: kondital: 25-40 ml O2 min-1 kg-1. En nedre grænse er fastsat for at danne en homogen gruppe. Forsøgspersonerne må ikke have deltaget i regelmæssig organiseret fysisk aktivitet de

seneste 2 år og må i forsøgsperioden ikke deltage i organiseret fysisk aktivitet uden for studiet. Imidlertid er de faste transportmønstre (cykling, gang) tilladt. Forsøgspersonerne må ikke have kendte sygdomme og desuden ikke have misbrug af alkohol, tobak, euforiserende stoffer eller tage daglig medicin. Som kvinde er det et krav at have en regelmæssig menstruation for at blive inkluderet i projektet. Det er ingen forhindring at deltage, hvis de kvindelige forsøgsdeltagere er på p-piller.

Statistiske overvejelser

Antallet af forsøgspersoner er valgt ud fra en forventning om fuldt dataset hos minimum 75% af deltagerne svarende til minimum 12 i hver gruppe, og standardafvigelser for effektmålene er fastsat svarende til de observerede forskelle i et tidligere studie på utrænede (Krustrup et al., 2009, 2010). Kondital og muskelmasse er valgt som primære effektmål.

Afgivelse af mundtlig information og indhentelse af samtykke

Efter at have udvist interesse modtager potentielle forsøgspersoner det skriftlige materiale (den vedlagte information til forsøgspersoner og folderen ”før du beslutter dig”). Herefter indkaldes forsøgspersonerne til samtale, hvor projektgruppens medlemmer supplerer den skriftlige information med mundtlig information om baggrunden for forsøget, formålet og de planlagte forsøgsdage. Desuden gennemgås information om de anvendte metoder samt risici og bivirkninger. Det understreges, at man når som helst kan trække sig ud af forsøget uden at angive en grund herfor. Forsøgspersonerne gøres opmærksom på, at de kan medbringe en bisidder til dette møde. Det endelige tilsagn og underskrift på samtykkeerklæringen indhentes efter mindst 2 dages betænkningstid. Ingen forsøgsrelaterede procedurer igangsættes før det informerede samtykke er indhentet.

Bivirkninger, risici og ulemper

Muskelbiopsier og blodprøver

For at kunne bestemme de muskulære effekter af træningen udtages i alt 2 prøver (hvile-biopsier) fra lårmuskulaturen. Ved udtagning af muskelbiopsier lægges først en lokalbedøvelse (med lidokain) af huden, hvorefter der laves et snit i huden på ca. 0,5 cm. Herefter udtages muskelbiopsierne, der hver vejer ca. 0,1 gram. Der kan føles et ubehag og i nogle tilfælde smerte, i det øjeblik biopsien tages. Der kan udføres arbejde/fysisk aktivitet straks efter muskelbiopsier. Der vil dog være ømhed i 2-4 dage efter udtagelse af muskelbiopsien svarende til et "trælår". Ved udtagning af biopsier er der en risiko for at sensoriske nerver beskadiges lokalt. Dette vil betyde at et lille område på huden føles sovende i op til 1 år efter forsøget. I sjældne tilfælde (<1/5000) kan en motorisk lokal nerve beskadiges. I så fald kan evnen til at aktivere en lille del af musklen mistes, men totalt set vil muskelfunktionen være helt upåvirket. Desuden er der en risiko for infektion i insertionsstederne, hvor muskelbiopsierne er taget igennem. Risikoen for infektion modvirkes via instruktion om behandling af biopsi-insertionsstederne. Blodprøverne på testdag 2 udtages fra et venflon anlagt i en armvene i albueledet. I forbindelse med fjernelse af kateterne kan der opstå en mindre blødning, der giver misfarvning af huden. For at mindske denne blødning vil indstiksområdet blive komprimeret efter udtagelsen (let tryk på stedet). Udtagelse af muskelbiopsier og blodprøver er rutine på forsøgslaboratoriet og er forløbet uden betydende komplikationer i mere end 25 år.

DXA–scanning

I forbindelse med DXA-scanning udsendes svage røntgenstråler, hvorved kroppens sammensætning af forskellige vævstyper måles. Den stråledosis, som forsøgspersoner får, er meget lille. Til sammenligning meget mindre end ved de fleste røntgenundersøgelser og svarer til én dags baggrundsstråling. Det gør ikke ondt (kan ikke mærkes) og undersøgelsen er ikke forbundet med ubehag. I forbindelse med DXA-scanningerne udsendes svage røntgenstråler, der giver en forøget kræftrisiko på 0,0001%, hvilket må anses for at være en minimal risiko.

Muskelfunktion

Den kan hos nogle personer forekomme svag-til-moderat muskelømhed kortvarigt (1-3 dage) efter testningen af maximal muskelstyrke, power og RFD. Vi har gennem de sidste 25 år udført mere end 5000 isokinetiske muskelstyrke tests, gennemført under iagtagelse af gennemprøvede og standardiserede opvarmnings- og testningsprocedurer, og har herunder aldrig oplevet hverken akutte eller subakutte muskel- eller ledskader.

Risiko for skader i forbindelse med den udførte træningsintervention

Desværre savnes i litteraturen nøjagtig dokumentation for skadesrisikoen ved motionshåndbold. Det er dog velkendt, at der er en markant højere skadesrisiko ved egentlige kampe (5-10 gange højere) end ved småspilskampe på motionsniveau. Håndbold er en kontaktsport, hvor der er en lille risiko for skader. Under træning, hvor spillerne kender hinanden anses risikoen for at være endnu lavere, hvorfor risikoen i dette projekt må anses for at være minimal. Alligevel understreges det, at muskelforstrækninger (i bl.a. baglår og forlår), seneskader og vridskader (i bl.a. ankler og knæ) også kan opstå under spiltræning. Ligesom der er en risiko for forstuvninger af fingrene i forbindelse med at gribe bolden. For at mindske risikoen for skader gennemføres inden hver træning en opvarmning. Forsøgspersonerne vil formentlig specielt i starten opleve en muskelømhed som følge af træningen, hvilket er helt normalt, men der opfordres til at gøre opmærksom på eventuelle gener i forbindelse med træningen.

Håndtering af biologisk materiale og personoplysninger

Der oprettes en forskningsbiobank på Institut for Idræt og Ernæring for produkter af de udtagne muskelbiopsier, blodprøver og fæces-prøver. Over hele projektperioden udtages samlet 2 muskelbiopsier af hver ~0,1g, ~80 ml blod, 40 ml urin og 50 g fæces. Muskelvæv, blodprøver, urin og fæces-prøver benyttes til en lang række analyser og der vil være ganske lidt overskydende biologisk materiale tilbage ved forsøgets afslutning. Overskydende biologisk materiale destrueres senest 5 år efter forsøgets afslutning. Oplysninger vedrørende forsøgspersonerne beskyttes efter lov om behandling af personoplysninger og sundhedsloven. Projektet anmeldes til Datatilsynet ved journalnummer 2013-54-0522. Hver enkelt

forsøgsperson vil efter projektafviklingen have mulighed for at få adgang til egne data samt gennemsnitsdata for hele gruppen. I den skriftlige deltagerinformation spørges om hvorvidt forsøgsdeltagerne ønsker adgang til egne forsøgsdata indtil 2 måneder efter projektafslutning. Alle data vil blive anonymiseret. Den enkelte deltager vil tillige, hvis det ønskes, få udleveret sine egne resultater

Initiering af projektet og eksterne projektmidler

Projektet er initieret af Peter Krustrup, Professor ved Institut for Idræt og Ernæring, Københavns Universitet. Der er opnået projektstøtte fra DHF (Dansk Hånbold Forbund) på 400.000 kr. Endvidere har DGI (Danske Gymnastik og idrætsforeninger) og EHF (det Internationale Håndbold Forbund) støttet projektet med henholdsvis 50.000 og 75.000 kr. Til sidst har Trygfonden støttet med 896.000 kr. Projektmidlerne er udbetalt og indsat på en projektkonto på Institut for Idræt og Ernæring og skal bruges til løn til projektmedarbejdere og indkøb af udstyr samt til muskel -og blodanalyser. Projektkontoen er underkastet offentlig revision. Der er ligeledes søgt støtte hos kulturministeriet. Hvis der opnås yderligere støtte, anmeldes dette til Videnskabs Etisk Komité, og der udleveres ny deltagerinformation til forsøgsdeltagerne. Der er ingen i projektgruppen, som har tilknytning til private virksomheder med interesser i projektet.

Vederlag til forsøgspersoner

Der gives et vederlag til forsøgspersonerne på 1000 kr. for deltagelse i projektet. Udbetaling foregår når man som forsøgsperson har afsluttet sin deltagelse. Beløbet er skattepligtigt. Udtræder en forsøgsperson inden forsøgets afslutning udbetales kun halvdelen af beløbet. Når man tager i betragtning at forsøgsdeltagerne gennemfører 8 testdage og deltager i op til 30 træningspas, samt at der udtages 2 muskelbiopsier fordelt over 2 dage, er beløbet tilpas af beskeden størrelse til, at der ikke sker en utilbørlig påvirkning af forsøgspersonerne.

Rekruttering af forsøgspersoner

Forsøgspersonerne rekrutteres primært via opslag på biblioteker, sociale net-sider som facebook, indkøbscentre og offentlige institutioner. Ligeledes annonceres i lokalaviser og gratisavis (MetroExpress). Opslags- og annoncetekst er identisk og er bilagt denne ansøgning.

Formidling af forsøgsresultater

Det er planen at projektets resultater skal formidles bredt. Dette sikres bl.a. gennem Center for Holdspil og Sundheds netværk, der tæller aktører i forskellige kommuner, idrætsforbund og interesseorganisationer. Undersøgelsens resultater præsenteres tillige ved videnskabelige konferencer og ved udgivelsen af videnskabelige artikler i 2015-2016. Både positive, negative og inkonklusive resultater vil blive offentliggjort.

Forsikringsforhold

Deltagerne i forsøget er omfattet af patientforsikringen.

Etiske overvejelser

Forsøget vil bidrage med vigtig viden til, hvorledes motionshåndbold ligesom motionsfodbold kan benyttes i forebyggelsen af livsstilsygedomme. De 2 træningsinterventioner (mænd og kvinder) anses for at være træningsforbedrende og generelt sundhedsfremmende for de rekrutterede forsøgspersoner. Hvis det viser sig til den indledende samtale -og undersøgelser, at forsøgspersonen ikke er rask, sendes forsøgspersonen til videre udredning, primært hos egen læge, og kan derfor ikke inkluderes i projektet. Hvad angår forsøgspersonerne i kontrolgruppen skal de bibeholde deres livsstil. Ligesom i interventionsgrupperne kan kun raske forsøgspersoner i kontrolgruppen deltage – personer med hypertension, hyperglykæmi, knogleskørhed og andre sygdomme sendes videre til specifik behandling. Der udbetales et beskedent vederlag for svie og smerte, som ikke anses for at bevirke en utilbørlig påvirkning af forsøgspersonerne. I forbindelse

med forsøget udtages i alt 2 muskelprøver fra lårmuskulaturen. Udtagelse af muskelbiopsier foregår under lokalbedøvelse af det ovenliggende hudområde. For forsøgspersonerne kan der føles et ubehageligt tryk i musklen ved udtagningen, samt ømhed 2-4 dage efter udtagningen, svarende til et "trælår". Desuden efterlader biopsitagningen små ar på huden, der typisk er næsten usynlige 1-2 år efter forsøget. Blodprøver (højest 40 ml blod per forsøgsrunde svarende til mindre end 2% af den samlede blodmængde) og muskelbiopsier bruges til at afklare projektets problemstillinger og anses ikke som noget etisk problem for en gruppe af raske 18-30 årige. Forsøget udføres i fuld overensstemmelse med Helsinki–deklarationen. Oplysninger vedrørende forsøgspersonerne beskyttes efter lov om behandling af personoplysninger og sundhedsloven. Eventuelt overskydende biologisk materiale vil blive destrueret senest 5 år efter forsøgets afslutning. Både positive, negative og inkonklusive resultater vil blive offentliggjort. Ingen i projektgruppen har relation til private virksomheder, der kunne have interesse i studiets resultater.

Referencer

Bangsbo J, Hansen PR, Dvorak J, Krustrup P. Recreational football for disease prevention and treatment in untrained men: a narrative review examining cardiovascular health, lipid profile, body composition, muscle strength and functional capacity. *Br J Sports Med* 2015;49:568–576.

Bradley C (1994). The Well-being Questionnaire. Kapitel 6, "Handbook of psychology and diabetes. A guide to psychological measurement in diabetes research and practice". Editor: Clare Bradley. Publisher: Harwood Academic Publishers, New York.

Craig CL, Marshall AL, Sjöström M, Bauman AE, Booth ML, Ainsworth BE, Pratt M, Ekelund U, Yngve A, Sallis JF, Oja P (2003). International Physical Activity Questionnaire: 12-country reliability and validity. *Med Sci.Sports Exerc* 35(8): 1381-1395.

Krustrup P, Nielsen JJ, Krustrup B, Christensen JF, Pedersen H, Randers MB, Aagaard P, Petersen AM, Nybo L, Bangsbo J (2009). Recreational soccer is an effective health promoting activity for untrained men. *Br J Sports Med.* 43(11): 825-831.

Krustrup P, Aagaard P, Nybo L, Petersen J, Mohr M, Bangsbo J (2010). Recreational football as a health promoting activity: a topical review. *Scand J Med Sci Sports 20*, suppl 1: 1-13.

Krustrup P, Randers MB, Andersen LJ, Jackman SR, Bangsbo J, Hansen PR (2013). Soccer improves fitness and attenuates cardiovascular risk factors in hypertensive men. *Med. Sci. Sports Exerc.* 45(3): 553-560.

Nielsen G, Wikman JM, Jensen CJ, Schmidt JF, Gliemann L, Andersen TR (2014). Helath promotion: The impact of beliefs of health benefits, social relations and enjoyment on exercise continuation. *Scand J Med Sci Sports:* 24(Suppl. 1): 66-75.

Oja P, Titze S, Kokko S, Kujala UM, Heinonen A, Kelly P, Koski P, Foster C. Health benefits of different sport disciplines for adults: systematic review of observational and intervention studies with meta-analysis. *Br J Sports Med* 2015;49:434–440.

Ottesen L, Jeppesen RS, Krustrup BR (2010). The development of social capital through football and running: studying an intervention program for inactive women. *Scand J Med Sci Sports* 20, suppl 1: 118–131.

Póvoas SC, Seabra AF, Ascensão AA, Maoalhães J, Soares JM, Rebelo AN (2012). Physical and physiological demands of elite team handball. *J strength Cond Res:* 26(12):3365-75

Rheinberg F, Vollmeyer R, Engeser S (2003). Die erfassung des Flow-erlebens. In: Stienmeier-Pelster J, Rheinberg F, eds. Diagnostik von Motivation und Selbstkonzept. Göttingen: Hogrefe. 261-279

Uth J, Hornstrup T, Schmidt JF, Christensen JF, Frandsen C, Christensen KB, Helge EW, Brasso K, Rørth M, Midtgaard J, Krustrup P (2014). Football training improves lean body mass in men with prostate cancer undergoing androgen deprivation therapy. *Scand J Med Sci Sports*, 24(S1): 105-112

Zigmond AS, Snaith RP (1983). The Hospital Anxiety and Depression Scale. *Acta Psychiatr Scand*: 67: 361-370.
