# Supplementary material for: Effects of small-sided recreational team handball training on mechanical muscle function, body composition and bone mineralization in untrained young adults—A randomized controlled trial
Source: PLoS One. 2020 Nov 18;15(11):e0241359. doi: 10.1371/journal.pone.0241359 (PMC7673568; doi:10.1371/journal.pone.0241359)
Supplement: S4 File — (PDF) [file pone.0241359.s007.pdf]

Journal number: H-15008361

Hovedprotokol_v2_29-06-2015

Study Protocol submitted to the Science Ethics Committee of Copenhagen,

The Scientifical Ethical Committee of the Region Hovedstaden

Regionsgården, Kongens Vænge 2, 3400 Hillerød

Research project about the health effects of recreational team handball exercise training

Background

There is a high incidence of lifestyle diseases in modern society, such as cardiovascular disease, type 2 diabetes and osteoporosis. Physical inactivity is one of the major risk factors for the development of lifestyle diseases. Recent research has shown that small-sided football exercise can act as effective and broad-spectrum prevention and treatment of lifestyle diseases, as documented by marked positive effects on cardiovascular health as well as on metabolic and musculoskeletal fitness (Krustrup 2010, 2013; Uth et al. 2014; Bangsbo et al. 2015). Notably, these effect were observed in a range of healthy and clinical populations, including untrained men and women of 20, 30, 50 and 70 years and patient groups with high blood pressure, type 2 diabetes and prostate cancer. At the same time, it was observed that small-sided recreational football training builds friendships and creates social relationships, which are important elements for optimizing participation and retention of physical activity (Nielsen et al. 2014; Ottesen et al. 2010). Thus, recreational football exercise has been recognized as a health promoting team sport activity that can improve the quality of life of both inactive healthy and ill groups (Oja et al. 2015).

The thesis of the present study is that recreational team handball will result in many of the same health qualities as recreational football exercise due to the involvement of intense intermittent work periods with many changes of direction and high musculoskeletal impact. A pilot study in men (+40 yrs) with previous handball experience showed that participants had a high intensity during training, comprable to that seen during recreational football training in the same age group (Póvoas et al. 2012). Furthermore, participants demonstrated positive effects on conditional and body composition after the training period (Póvoas et al. 2012). In view of the many health-promoting and social elements that are involved in recreational football exercise, it becomes relevant to investigate recreational team handball exercise and its potential contribution to a healthier lifestyle among people who are inactive or otherwise do not participate in regular physical activity.

Objective

The aim of this study is to investigate the fitness and health effects of participating in recreational team handball for 12 weeks for untrained men and women aged 18-30 years.

Material and Methods

32 healthy untrained men (VO_{2max}: 30-45 ml O2 min-1 kg-1) and 32 healthy untrained women (VO_{2max}: 25-40 ml O2 min-1 kg-) aged 18-30 years will be recruited to complete 12 weeks in either (a) a group

engaged in regular recreational team handball for 12 wks or (b) a control group that continues their normal lifestyle. The study is a randomized controlled design. Subjects are randomized after stratification for VO_{2max} and fat percentage into the 2 groups mentioned above, completing the 12 weeks of team handball training or continuing their inactive lifestyles. The male and female subjects should not be included in the same training group or control group. Therefore, there will be 2 training groups (one male and one female) and 2 control groups (one male and one female) with 16 subjects in each group.

Training

The training will consist of 2-3 weekly workouts of approx. 70 min duration for 12 weeks. The first 15 minutes are set aside for warm-up, after which 4 * 10 min are played with a 3 min break between each game. Control participants are instructed to make no changes to their normal lifestyle. The training sessions will take place at the Department of Nutrition and Exercise Science, Nørre Alle 53, Copenhagen N, Denmark.

Testing

Testing is performed before the training period (1st test round) and after 12 weeks of intervention period (2nd test round). These Testdays aim to investigate subjects cardiovascular, metabolic and musculoskeletal fitness as well as identify a number of significant health parameters including blood pressure, vascular function, blood sugar, cholesterol, fat percentage and bone mineralization. These variables will be obtained on 4 seperate Testdays, each seperated by at least 48 hours.

Testday 1

Testday 1 will take place at Department of Nutrition and Exercise Science, Universitetsparken 13, 2300 Copenhagen Ø. This Testday will be started with measurement of blood pressure to support the subject is healthy. At a scrrening day prior to Testday 1 subject will be approved to participate in the study, based on the information given to the well-trained staff (Professor, Peter Krustrup, Human Physiologist and PhD student, Therese Hornstrup , Human Physiologist). The blood pressure measurement is followed by a treadmill test consisting of 2 bouts of 5 min running at 6.5 and 8 km/h, respectively, followed by a maximum running test consisting of 4 min at 8 km/h, then increasing the running speed by 1 km/h each minute to exhaustion. Pulmonary ventilation and the oxygen composition of inhaled and exhaled air are measured to determine VO_{2max}. Testday 1 will last approximately 1.5 hours.

Testday 2

Testday 2 will take place at both the Frederiksberg and Østerbro departments of the Department of Nutrition and Exercise Science. The day will start with a fasting blood sample taken from an arm vein to measure blood sugar, long-term blood sugar, insulin, hematocrit value, cholesterol, inflammation markers and bone markers (~ 40 ml blood). This is followed up by a questionnaire survey that participants complete on a computer. The questionnaire consists of questions about socio-economic status, activity level (International Physical Activity Questionnaire: Craig et al. 2003), mental health (Hospital Anxiety Depression Scale: Zigmond & Snaith, 1983) well-being (Bradley 1994) and motivation for sport in general

(Sport Motivation Scale : Pelletier et al. 1995). All of these questionnaires have been validated in Danish or in the English form from which they are translated and are suitable for the target group. Next, a full-body DXA scan is performed to determine fat percentage, muscle and bone mass, and bone density. After 30 minutes of rest, 5 consecutive blood pressure measurements are performed, followed by an endothelial function test, which is a non-invasive method for measuring vessel function. Vascular function is measured in supine position using the peripheral artery tonometry (PAT) method. The PAT measures the pulse wave amplitude during reactive hyperemia using a fingerpletysmograph. Finally, a muscle sample is taken from the thigh muscle under anesthesia. This is used for histochemical determination of fiber type distribution and fiber type specific capillarization, enzymatic and metabolic analysis. Testday 2 lasts 3 hours.

Testday 3

Testday 3 will take place at the Department of Nutrition and Exercise Science, Universitetsparken 13, 2300 Copenhagen Ø and Bispebjerg Hospital, Building 10 ground floor, 2400 Copenhagen NV. Echocardiography: Cardiac function will be measured by advanced tissue Doppler echocardiography to study the contraction and relaxation patterns of muscle tissue. 2D strain analyzes are used which allow angle independent analyzes of both longitudinal and radial contraction patterns and in addition, circumferential movement patterns are assessed, and thus the twist itself contributed to the global myocardial function. Investigations are made of correlation between radial and longitudinal contraction, relationship between twist (circumferential rotation at apex and base, relationship between twist and diastolic function, relationship between twist, diastolic function and atrial dimension, and changes under load. Measurements will take place while the subject is in the supine position of Muscle function: Following a standardized warm-up cycle on a ergometer cycle, a Nottingham power rig test is performed to measure maximum leg muscle power, postural sway balance test on one leg (static and frontal hop respectively) and finally measurement of maximum dynamic (concentric) and isometric (static)) muscle strength of the knee extensor muscle (dominant legs) in an isokinetic dynamometer, (KinCom) including measurement of maximum explosive muscle strength (Rate of Force Development: RFD).

Testday 4

Testday 4 will take place in connection with the first training session at the Department of Nutrition and Exercise Science, Nørre Alle 53, Copenhagen N. A Yo-Yo Interval Endurance Test - Level 1 will be conducted. This test includes 2x20 meters runs back and forth between top marks. After each trip, a 5 s break is held. The speed during the runs is gradually increased by beep signals on a CD. First time the run is not compeated, a warning is received. Second time the test is over. The running distance (level) is recorded as a test result.

In addition to the above testdays, a heart rate belt and a GPS meter will be provided to measure intensity and workout during selected training sessions. In these selected training sessions, a questionnaire on the severity of the training session, soreness after exercise, the experience of flow during the training (Flow Kurz Scale: Rheinberg et al. 2003), the experience of the enjoyment of the training (Kendzierski &

DeCarlo, 1991), and motivation for handball (Sport Motivation Scale: Pelletier et al. 1995) is also filled out.

For study participants volunteering for a subprocedure, there will be a opportunity to deliver a fecal and urine test before and after the intervention to characterize the gut bacterial composition and the functional potential of the gut. The samples are collected in the subjects own homes, and they are provided with the necessary equipment to perform the procedure as well as a thorough guide to how the samples should be handled in relation to storage and hygiene. The samples are frozen at -18 degrees and staff from the University of Copenhagen will collect the samples within a week and transport them to the University for further storage. All samples are collected according to standard procedure for subsequent standardized DNA and RNA extraction.

Recruitment of study participants, inclusion and exclusion criteria

Study participants should be healthy yet untrained men: VO_{2max}: 30-45 ml O2 min-1 kg-1 and women: VO_{2max}: 25-40 ml O2 min-1 kg-1. A lower limit in VO_{2max} is applied to ensure a homogeneous group. The subjects must not have participated in regular organized physical for the past 2 years and may not participate in organized physical activity outside the study during the trial period. However transport (cycling, walking) are allowed. The subjects must not have any known diseases and, moreover, not abuse alcohol, tobacco, euphoric drugs or take daily medication. As a woman it is a requirement to have a regular period to be included in the project. Participation is allowed if the female study participants are on birth control pills.

Statistical considerations

The number of subjects was chosen based on the expectation of a full dataset of at least 75% of the participants corresponding to a minimum of 12 in each group, and standard deviations for the power measures were determined according to the observed differences in a previous study on untrained (Krustrup et al., 2009, 2010). VO_{2max} and muscle mass are chosen as the primary effect objectives.

Oral and written information, collection of consent

After showing interest, prospective subjects will receive written material about the project (the enclosed information for the subjects and the leaflet "before you decide"). Subsequently, the subjects are invited to a conversation where the project group members will supplement the written information with oral information about the background of the experiment, the purpose and the planned trial days. In addition, information on the methods used, risks and side effects are reviewed. It is emphasized that the study participants can withdraw from the study at any time without stating a reason. The subjects are informed that they may bring a bystander to this meeting. The final commitment and signature on the consent declaration is obtained after at least 2 days of reflection. No trial-related procedures are initiated until the informed consent is obtained.

Side effects, risks and disadvantages

Muscle biopsies and blood tests

In order to determine the muscular effects of the training, a total of 2 samples (biopsies) will be taken from the thigh muscles. When removing muscle biopsies, local anesthetic (lidocaine) is applied to the skin, after which an incision is made into th skin of approx. 0.5 cm. Subsequently, the muscle biopsies, each weighing approx. 0.1 grams. There may be discomfort and, in some cases, mild-to-moderate pain in relation to the biopsy collection procedure. Exercise/physical activity can be performed immediately after sampling of muscle biopsies. However, there will be soreness for 2-4 days after taking the muscle biopsy. When biopsies are taken, there is a low risk that sensory nerves are locally damaged. This will mean that a small area of the skin may feel dormant or insensive for up to 1 year after the experiment. In rare cases (<1/5000), a motor local nerve can be damaged. In that case, the ability to activate a small part of the muscle may be lost, but overall the muscle function will be completely unaffected. In addition, there is a risk of infection in the insertion sites where the muscle biopsies are taken. The risk of infection is counteracted through instruction on the treatment of the insertion sites. The blood samples on testday 2 are taken from a vein flap placed in an arm vein in the elbow joint. In connection with the removal of the catheters, minor bleeding may occur, causing discoloration of the skin. To reduce this bleeding, the insertion area will be compressed after removal (light pressure on site). Muscle biopsies and blood tests are routine in the laboratory and have been without significant complications for more than 25 years.

DXA-scans

In connection with DXA-scans, small doses of X-rays are emitted, which measures the body’s composition of different tissue types. The radiation dose is very small, corresponding to one day's natural background radiation, equivalent of an increased cancer risk of 0.0001%. The DXA assessment is not associated with any discomfort.

Muscle function

After testing for maximum muscle strength, power and rapid force capacity (rate of force development: RFD) participants may briefly (1-3 days) experience mild-to-moderate muscle soreness. Over the past 25 years we have performed more than 5000 isokinetic muscle strength tests, conducted in accordance with proven and standardized warm-up and testing procedures, and we have never experienced either acute or subacute muscle or joint injuries.

Risk of injury in correlation to the team handball training

Literature lacks accurate documentation of the injury risk of exercise handball. However, it is well known that there is a significantly higher risk of injury in actual matches (5-10 times higher) than in friendly small-sided match plays for recreational purpose. Handball is a contact sport where the risk of injury present. During training between acquaintances the risk is considered to be even lower, so the risk in this project must be considered minimal. Nevertheless, it is emphasized that muscle sprains (in hamstrings and quadriceps), tendon injuries and torsional injuries (in the ankles and knees, among others) can occur during training. Just as there is a risk of sprains of the fingers associated with catching the ball. To reduce the risk of injury, a warm-up is performed before each session of match play. During the initial training

phase (wks 1-2) study participants will probably experience muscle soreness as a result of the training, which is quite normal, but are encouraged to point out any muscle irritation associated with the training.

Handling of biological material and personal information

A biobank is set up at the Department of Nutrition and Exercise Science for muscle biopsies, blood samples and feces samples. Over the entire project period, a total of 2 muscle biopsies are taken from each ~ 0.1g, ~ 80ml blood, 40ml urine and 50g faeces. Muscle tissue, blood samples, urine and feces samples are used for a wide range of analyzes and there will be very little excess biological material left at the end of the experiment. Excess biological material is destroyed within 5 years of the end of the experiment. Information regarding the subjects is protected under the Personal Data Processing Act and the Health Act. The project is reported to the Danish Data Protection Agency by journal number 2013-54-0522. After completion of the project, the study participant will have the opportunity to access his own data and average data for the whole group. The study participants has given information regarding if they want their data after the study has been completed. All data will be anonymized.

Initiation of the project and external project funds

This project is initiated by Peter Krustrup, Professor at the Department of Nutrition and Exercise Science. Finacial support has been given by DHF (Danish Handball Federation) with DKK 400,000. Furthermore, DGI (Danish Gymnastics and Sports Associations) and EHF (the International Handball Federation) have supported the project with DKK 50,000 and 75,000 respectively. Finally, Trygfonden has supported with DKK 896,000. The project funds have been paid out and deposited into a project account at the Department of Sport and Nutrition and will be used for salaries for project employees and the purchase of equipment as well as for muscle and blood analyzes. The project account is subject to public audit. Financial support has also been applied for at the Ministry of Culture. If additional support is obtained, this will be reported to the Science Ethics Committee and new participant information will be provided to the study participants. There is no one in the project group who is affiliated with private companies with interests in the project.

Compensation of test subjects

Compensation of DKK 1000 will be given to the subjects after participation in the project. Payment is paid when the participant has completed his or her participation. The compensation is subject to tax. If a study participant resigns before the end of the trial, only half of the amount will be paid. Taking into account that the test participants complete 8 Testdays and participate in up to 30 training sessions, and they have 2 muscle biopsies taken, the amount is sufficiently small to prevent undue influence on the subjects.

Recruitment of study participants

Study participants will be recruited primarily through poster notifications in public and university libraries, social medias such as facebook, shopping malls and public institutions. There will also bee advertised in local and free newspapers (MetroExpress). Text are identical and are attached to this application.

Distribution of study results

The plan is to distribute the study results widely. This is ensured, among other things through Center for Team Games and Health Network, which includes players in various municipalities, sports federations and interest organizations. The results of the study are also presented at scientific conferences and the publication of scientific articles in 2015-2016. Both positive, negative and inclusive results will be published.

Insurance

Study participants are covered by the the Danish patient insurance.

Ethical considerations

This study will provide important knowledge about to which extent small-sided team handball training may be useful in the prevention of lifestyle diseases in untrained young adults. The training intervention is considered to be generally health-promoting for the recruited subjects. If it appears from the initial conversation and studies that the subject is not well, the subject is sent for further investigation, primarily from his own physician, and therefore cannot be included in the project. As for the subjects in the control group, they must maintain their lifestyle. As in the intervention groups, only healthy subjects can participate - people with hypertension, hyperglycemia, osteoporosis and other diseases are passed on for specific treatment. A modest compensation is paid which is considered not to have any influence on the subjects motivation for participation in the study. In correlation to the study a total of 2 muscle samples are taken from the thigh muscle for each male study participant. Muscle biopsies are taken out under local anesthesia of the surrounding skin area. For the subjects, uncomfortable pressure can be felt in the muscle at the time of removal, as well as soreness 2-4 days after obtainment of biopsie. In addition, biopsy sampling leaves small scars on the skin, which is typically almost invisible 1-2 years after the experiment. Blood samples (maximum 40 ml blood per test round corresponding to less than 2% of the total blood volume) and muscle biopsies are used to clarify the project's aim and are not considered an ethical problem for a group of healthy 18-30 year olds. The experiment is carried out in full compliance with the Helsinki Declaration. Information regarding the subjects is protected under the Personal Data Processing Act and the Health Act. Any excess biological material will be destroyed within 5 years of the end of the experiment. Positive, negative and inclusive results will be published. No one in the project group is related to private companies that could be interested in the results of the study.

References

Bangsbo J, Hansen PR, Dvorak J, Krustrup P. Recreational football for disease prevention and treatment in untrained men: a narrative review examining cardiovascular health, lipid profile, body composition, muscle strength and functional capacity. Br J Sports Med 2015;49:568–576.

Bradley C (1994). The Well-being Questionnaire. Kapitel 6, "Handbook of psychology and diabetes. A guide to psychological measurement in diabetes research and practice". Editor: Clare Bradley. Publisher: Harwood Academic Publishers, New York.

Craig CL, Marshall AL, Sjöström M, Bauman AE, Booth ML, Ainsworth BE, Pratt M, Ekelund U, Yngve A, Sallis JF, Oja P (2003). International Physical Activity Questionnaire: 12-country reliability and validity. Med Sci.Sports Exerc 35(8): 1381-1395.

Krustrup P, Nielsen JJ, Krustrup B, Christensen JF, Pedersen H, Randers MB, Aagaard P, Petersen AM, Nybo L, Bangsbo J (2009). Recreational soccer is an effective health promoting activity for untrained men. Br J Sports Med. 43(11): 825-831.

Krustrup P, Aagaard P, Nybo L, Petersen J, Mohr M, Bangsbo J (2010). Recreational football as a health promoting activity: a topical review. Scand J Med Sci Sports 20, suppl 1: 1-13.

Krustrup P, Randers MB, Andersen LJ, Jackman SR, Bangsbo J, Hansen PR (2013). Soccer improves fitness and attenuates cardiovascular risk factors in hypertensive men. Med. Sci. Sports Exerc. 45(3): 553-560.

Nielsen G, Wikman JM, Jensen CJ, Schmidt JF, Gliemann L, Andersen TR (2014). Helath promotion: The impact of beliefs of health benefits, social relations and enjoyment on exercise continuation. Scand J Med Sci Sports: 24(Suppl. 1): 66-75.

Oja P, Titze S, Kokko S, Kujala UM, Heinonen A, Kelly P, Koski P, Foster C. Health benefits of different sport disciplines for adults: systematic review of observational and intervention studies with meta-analysis. Br J Sports Med 2015;49:434–440.

Ottesen L, Jeppesen RS, Krustrup BR (2010). The development of social capital through football and running: studying an intervention program for inactive women. Scand J Med Sci Sports 20, suppl 1: 118–131.

Póvoas SC, Seabra AF, Ascensão AA, Maoalhães J, Soares JM, Rebelo AN (2012). Physical and physiological demands of elite team handball. J strength Cond Res: 26(12):3365-75

Rheinberg F, Vollmeyer R, Engeser S (2003). Die erfassung des Flow-erlebens. In: Stienmeier-Pelster J, Rheinberg F, eds. Diagnostik von Motivation und Selbstkonzept. Göttingen: Hogrefe. 261-279

Uth J, Hornstrup T, Schmidt JF, Christensen JF, Frandsen C, Christensen KB, Helge EW, Brasso K, Rørth M, Midtgaard J, Krustrup P (2014). Football training improves lean body mass in men with prostate cancer undergoing androgen deprivation therapy. Scand J Med Sci Sports, 24(S1): 105-112 Zigmond AS, Snaith RP (1983). The Hospital Anxiety and Depression Scale. Acta Psychiatr Scand: 67: 361-370.
